# Draft Genome Sequence of MKD8, a Conjugal Recipient *Mycobacterium smegmatis* Strain

**DOI:** 10.1128/genomeA.00148-13

**Published:** 2013-04-25

**Authors:** Todd A. Gray, Michael J. Palumbo, Keith M. Derbyshire

**Affiliations:** Division of Genetics, Wadsworth Center, New York State Department of Health, Albany, New York, USAa;; Division of Translational Medicine, Wadsworth Center, New York State Department of Health, Albany, New York, USAb;; Department of Biomedical Sciences, University at Albany, State University of New York, Albany, New York, USAc

## Abstract

We report an annotated draft genome sequence of the *Mycobacterium smegmatis* strain MKD8. This strain acts as a recipient during conjugation with the reference *M. smegmatis* strain mc^2^155. While the genomes of the two strains are colinear and have similar sizes, extensive genome-wide sequence variation suggests rich diversity within the *M. smegmatis* clade.

## GENOME ANNOUNCEMENT

Mycobacteria are a biologically diverse group of bacteria that range from obligate pathogens, including *Mycobacterium tuberculosis*, to free-living saprophytes, such as *Mycobacterium smegmatis*. *M. smegmatis* is the model organism for all mycobacterial research because it is nonpathogenic, relatively fast-growing, genetically facile, and well suited for high-throughput analyses (1, 2). Natural isolates of *M. smegmatis* participate in a novel form of conjugation, termed distributive conjugal transfer, in which chromosomal DNA segments are transferred from a donor strain to a recipient strain (3, 4) and generate transconjugant progeny with highly mosaic genomes (T. A. Gray, J. Krywy, J. Harold, M. J. Palumbo, and K. M. Derbyshire, submitted for publication). Our standard experimental conjugation system pairs the widely used laboratory strain *M. smegmatis* mc^2^155 (a donor) with an independent strain, *M. smegmatis* MKD8 (a recipient). MKD8 is a spontaneous streptomycin-resistant subclone of *M. smegmatis* mc2874 (*lysA ept* [5]), derived from the original isolate, *M. smegmatis* PM5 (6). Determining the chromosomal sequence of this strain is necessary to accurately analyze the mosaic transconjugant genomes, identify those genes that distinguish donor and recipient functions, and begin to explore the rich genetic diversity of *M. smegmatis* strains.

The genomic sequence of *M. smegmatis* MKD8 was compiled from the data from three approaches: an initial 454 library, an Illumina ~250-bp paired-end library, and a second 454 approach yielding a ~3-kb paired-end library to join some of the repeat-separated contigs. Aggregate reads were assembled *de novo* using Celera Assembler with the Best Overlap Graph (CABOG) (7) and Velvet (8). The two scaffolds were separated by the two rRNA loci present in *M. smegmatis*. The assembled draft genome was annotated using the Annotation Engine service (http://ae.igs.umaryland.edu/cgi/index.cgi) and visualized using the Manatee genome curation and browsing tool (http://manatee.sourceforge.net).

The MKD8 genome is 7,092,137 bp long, with an overall G+C content of 67.3%, similar to that of mc^2^155 (6,988,209 bp; GenBank accession no. NC_008596). Whole-genome comparisons by Mauve (9) showed an overall colinearity between the two strains but significant sequence divergence (1.6% overall single-nucleotide polymorphism [SNP] frequency and 649 indels of >19 bp). One notable difference is that MKD8 lacks the 55.2-kb genome duplication present in mc^2^155 (*msmeg1002* to *msmeg1058*). Subsets of these SNPs and indels are likely responsible for the many phenotypic differences displayed by these two strains (e.g., in donor and recipient activities, colony morphology, phage susceptibility, biofilm formation, and streptomycin resistance).

Conjugation in mycobacteria generates progeny that are genetic mosaics of the parental strains. Since multiple independent strains of *M. smegmatis* are conjugationally active (3, 10), mosaic transconjugant genomes could be generated with genes that are lost, acquired, replaced, or blended. Such mosaic transconjugant genotypes might combine phenotypes, or potentially create new phenotypes, to promote the colonization of different environmental niches or hosts. Therefore, transconjugants generated today may become the extant mycobacteria of tomorrow. Establishing the reference genomes of conjugationally active mycobacteria is essential for recognizing the products of conjugation, estimating the gene flow through the community, and identifying the genetic basis of the phenotypes that drive the evolution of mycobacteria. The determination of the MKD8 sequence is an important first step in that direction.

### Nucleotide sequence accession number. 

The assembled and annotated draft genome sequence of *M. smegmatis* MKD8 has been deposited in GenBank under the accession no. CM001762.
